# The Application of Biological Feedback in the Rehabilitation of Patients after Ischemic Stroke

**DOI:** 10.3390/s22051769

**Published:** 2022-02-24

**Authors:** Marzena Mańdziuk, Marlena Krawczyk-Suszek, Ryszard Maciejewski, Jerzy Bednarski, Andrzej Kotyra, Weronika Cyganik

**Affiliations:** 1 Medical College, University of Information Technology and Management in Rzeszow, 2 Sucharskiego Str., 35-225 Rzeszow, Poland; mkrawczyk@wsiz.edu.pl (M.K.-S.); wcyganik@wsiz.edu.pl (W.C.); 2 Department of Human Anatomy, Medical University of Lublin, 19 Chodzki Str., 20-093 Lublin, Poland; ryszard.maciejewski@umlub.pl (R.M.); jerzy.bednarski@umlub.pl (J.B.); 3 Department of Electronics and Information Technology, Faculty of Electrical Engineering and Computer Science, Lublin University of Technology, 38a Nadbystrzycka Str., 20-618 Lublin, Poland; a.kotyra@pollub.pl

**Keywords:** balance-trainer, biofeedback, rehabilitation, stroke

## Abstract

Balance disorders are the main concern for patients after an ischemic stroke. They are caused by an abnormal force on the affected side or paresis, which causes uneven loading and visuospatial disorders. Minimizing the effects of stroke is possible through properly conducted rehabilitation. One of the known ways to achieve this objective is biological feedback. The lack of proper muscle tone on one side of the body is manifested by the uneven pressure of the lower extremities on the ground. The study and control groups were composed of two equal groups of 92 people each, in which the same set of kinesiotherapeutic exercises were applied. Patients in the study group, in addition to standard medical procedures, exercised five days a week on a Balance Trainer for four weeks. The examination and training with the device were recorded on the first day of rehabilitation, as well as after two and four weeks of training. The assessment was performed using the following functional tests and scales: Brunnström, Rankin, Barthel, Ashworth, and VAS. Patients in the control group started exercising on the Balance Trainer two weeks after the first day of rehabilitation using traditional methods. The study results reveal statistically significant reductions in the time the body’s center of gravity (COG) spent in the tacks, outside the tracks and in the COG distance, lower COG excursions in all directions. Post-stroke patients that received biofeedback training presented significantly better results than patients that did not receive such training.

## 1. Introduction

Stroke is the most common cause of adult disability and the second most common reason for death in developed countries, causing a major medical and social problem [1]. Worldwide, between 4.6 and 5.7 million people die from stroke each year [1,2]. Predictions covering the period until 2030 indicate an increase in the number of people dying due to stroke. The number of people dying from stroke is expected to rise by 2030 due to population ageing and economic changes in underdeveloped countries [3,4,5].

The most common concerns among stroke patients are balance disorders and problems maintaining an upright body position. The imbalance can be influenced by the nervous system using feedback, movement pacing, and teaching phases of motor control [6,7]. This provides the opportunity to influence existing and newly formed neuronal pathways using auditory and visual stimuli. Recently, many games and systems have been developed based on the use of biological feedback to train the body balance of post-stroke patients [8,9,10,11]. The current balance rehabilitation of stroke patients is based on the use of conventional exercises, but also modern technologies, such as biofeedback and virtual reality [9,10,12,13,14,15].

Virtual reality is a technology allowing the development of a computer-generated environment in which one can interact with any object as well as perform movement tasks [16]. In contrast, biofeedback (BF) increases awareness of movement and/or function, and has been used in rehabilitation for over fifty years to restore normal movement patterns [16]. Using modern technology, specific body parts can be trained more effectively, and training can be adjusted to the individual abilities of stroke patients. Thanks to exercises using virtual reality, the patient can train more intensively as the body is forced to do more work. This leads to the stimulation of the reaction capabilities and body balance [17,18,19].

Many authors emphasize the positive effects on trunk control, balance, and gait ability [11,13,20,21,22,23,24,25,26,27]. 

These authors show the remarkable effectiveness of balance training to develop and improve the sense of balance [28,29]. The combination of individually guided rehabilitation with biofeedback exercises results in five times higher relative lower limb pressure on the side with paresis compared to the group following the same program without biofeedback [30]. The Balance Trainer uses the phenomenon of biological feedback (biofeedback), which ensures that patients with sensory–motor disorders regain the ability to assess various physiological reactions more effectively, and to self-control their motor response [31]. These researchers used version II of the Balance Trainer device. Currently, there are upgraded devices (version IV of the device is available on the market) that have new software that allows rehabilitation in virtual reality while playing games using 3D technology. The THERA-Trainer product line allows for comprehensive rehabilitation of patients from sitting to active standing and balance training to gait re-education.

There are several publications in the literature that demonstrate the results of training with the use of the Balance Trainer in a group of patients after stroke [32,33]. In the present study, various timings of biofeedback therapy were applied in the study and control groups; exercise schedule and methods of patient evaluation are the focal schemes in this study for managing patients with this disease. 

### Biological Feedback

Biofeedback is a method of providing a person with information regarding the state of their body using various devices in the form of light, sound, or other signals. Biofeedback is a technique using electronic apparatus that allows the patient to learn how to change the physiological function of the body to achieve improvements in health and in the efficiency and effectiveness of a specific bodily function [18]. Biofeedback is also defined as a body mind training method that helps patients achieve awareness and control over physiological processes, such as respiration, heart rate, muscle tone, skin temperature, electrodermal response, and blood pressure, and during hemoencephalography recording [34,35].

The central nervous system (CNS) plays a major role in the biofeedback phenomenon by receiving and analyzing stimuli originating from proprioceptors. The primary regulation of all physiological functions of the body controlled by the CNS is a physiological biological feedback mechanism based on the neuromechanism of neural reflexes. If there is a disruptive arbitrary movement, an extrinsic sensory feedback loop can be used to teach the restoration of the correct movement pattern. The above method can only be applied to patients that fully and consciously cooperate with the therapist [36,37]. All nerve cell activity involves a change in action potential accompanied by the release of a transmitter substance [38]. There is a change in the membrane potential and the so-called sprouting process, which results in a biochemical and anatomical change. This forms the basis of synaptogenesis (neuromodulation). Strong and systematic stimulation generates reinforcement and contributes to the formation of new connections (long-term potentiation, LTP), whereas weak stimulation does not initiate the long-term reinforcement of the so-called long-term memory, and subthreshold values may even impair it. The following two basic systems of brainwave generation play an important role in the process of neuromodulation: the thalamocortical system, where the processing and selecting operations of stimuli take place; and the hippocampus, together with the frontal lobes and thalamus, where the regulation of attention, concentration, and memory takes place. The two systems remain dependent on each other and form what is known as a feedback loop (excitatory, inhibitory) that depends on the activity of the cortex. When dysregulation occurs within the activity of these systems, an alternative corrective measure is to restore their stability. Biofeedback based on autoregulatory training techniques increases this stability and restores internal consistency, and through systematic interactions causes the formation of new neuronal circuits [39]. Biofeedback finds its application in the rehabilitation of patients with neurological disorders, including after stroke. Using biofeedback exercises, locomotion and gait function, limb loading and balance, coordination, and defensive responses can be improved. Intensive training is indicated in rehabilitation after stroke [40]. A key element of biofeedback is the control of the body’s center of gravity excursions while the exerciser is standing on a static–dynamic parapodium, measuring COG excursions with changes in position and limb movements during the performed exercise. A point image is used, showing the body’s center of gravity, which allows the patient to attempt to modify the alignment of the trunk and lower extremities and, as a result, to correct their posture. This mechanism increases the patient’s motivation, stimulates proprioceptive analysis, and facilitates communication with the therapist [41]. Patients re-learn certain movement actions needed to produce the intended effect. The best movement should be accurately repeated to achieve the effect of re-learning that movement. It is important that the individual performs and memorizes a series of movements resembling the perfect movement, continually striving for the best effect by correcting the execution based on practiced movement experience [42].

The purpose of this study is to evaluate the effectiveness of biological feedback training, using the Balance Trainer, on patients after cerebral ischemic stroke.

**Hypothesis** **1:** *The use of Balance Trainer exercises affects the conscious control of the standing posture of stroke patients with hemiparesis.*

**Hypothesis** **2:** *Training with the Balance Trainer reduces spasticity.*

**Hypothesis** **3:** *Training with the Balance Trainer improves lower limb function.*

**Hypothesis** **4:** *Training with the Balance Trainer influences an increase in the degree of independence.*

**Hypothesis** **5:** *Training with the Balance Trainer influences a decrease in disability.*

## 2. Materials and Methods

### 2.1. Study Group

The research was conducted among patients treated in the Rehabilitation Clinic with the Early Neurological Rehabilitation Ward of the Queen St Hedwig’s Hospital in Rzeszów. The basis for the implementation of the study was the decision of the Bioethics Committee at the Medical University of Lublin, Resolution No. KE-0254/45/2018.

Participation criteria for both groups of patients were: a history of ischemic stroke, one stroke in a lifetime, no more than six months after the stroke, existence of hemiparesis, coherent communication and response to commands, stabilized cardiorespiratory parameters allowing at least passive upright standing, and consent of the patient to participate in the experiment.

The exclusion criteria for both groups of patients from the study were: other diseases of the central nervous system, a history of an injury in the lower limbs within the last three months, a visual impairment preventing the tracking of the center of gravity (COG) movements on the monitor, other medical indications.

Finally, 92 patients from the study group (38 women and 54 men) and 92 patients from the control group (41 women and 51 men) were enrolled in the study. The age of the study group ranged between 48 and 76 years, while the age of the control group ranged between 49 and 73 years. The average BMI of the study group was 27.22 ($\frac{\mathrm{kg}}{m^{2}}$), and that of the control group was 26.20 ($\frac{\mathrm{kg}}{m^{2}}$). The figures of the most important descriptive statistics are included in Table 1.

In the study group, 56 patients had right-sided paralysis and 36 patients had left-sided paralysis. In the case of the control group, the majority (71 patients) had left-sided paralysis, while 21 patients had right-sided paralysis.

### 2.2. Methodology of the Study

**Figure 1:**
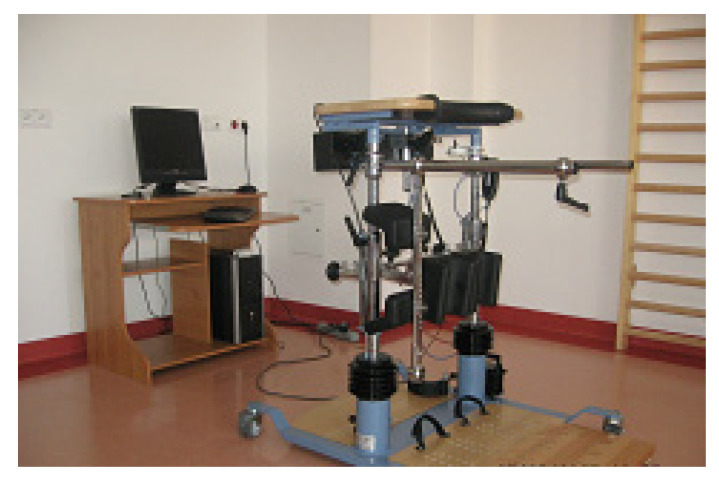
Balance Trainer (own sources).

The same set of kinesiotherapeutic exercises were used in both groups, as follows: balance exercises, motor coordination, individual whole-body training (conducted using the Bobath and PNF methods), breathing exercises, and passive or active verticalization, depending on the functional status of the patients. In addition, all patients underwent psychotherapy and neurologopedics, conducted every day for half an hour at a time. The patients in the study group exercised on a Balance Trainer (Figure 1) five days a week for four weeks, in addition to the standard medical procedures. Patients in the control group started exercising on the Balance Trainer two weeks after the first day of rehabilitation using traditional methods. A delay occurred because the Balance Trainer could only be used for two hours a day, which is not enough time for a large number of patients. The doctor decided which patient would use the Balance Trainer first. The members of the research team could not influence the doctor’s decisions. Both groups of patients were evaluated using the following functional scales: Brunnström, Rankin, Barthel, Ashworth, and VAS, the results of which were documented three times.

Before the study, each patient had the opportunity to familiarize themselves with the equipment and its functioning (performing one trial of movements); they were informed about the methodology, purpose of the study and the anonymity of the study. Each patient gave written consent to participate in the research and was informed about the possibility of withdrawal from the study at any stage of its duration. The patients performed all exercises on the Balance Trainer device in accordance with the methodology described in the device manual. To determine the studied parameters, the following actions were performed:

- Movement on a horizontal track (from right-to-left);
- Movement on a horizontal track (from left-to-right);
- Vertical trajectory (from back-to-front);
- A tilt test (COG).

The recording of each parameter was performed three times; on the admission of the patient to the ward, after two weeks of rehabilitation, and after four weeks of the patient’s hospitalization. After each training session, a tilt test without visual control was performed to assess the tilting of the body’s center of gravity from the vertical.

The objective of the analysis was to compare three dependent measurements of individual parameters, measured using the Balance Trainer device, for the analyzed groups, and to compare measurements of the two unrelated groups, i.e., control and study.

### 2.3. Data Analysis

The analysis of dependent variables for more than two variables was performed using Friedman’s ANOVA test, the comparative analysis of two independent groups was performed using the Mann–Whitney U test. The normality of the distributions of quantitative variables was checked using the Shapiro–Wilk test. The absence of normal distribution of quantitative variables conditioned the use of non-parametric statistics. 

Statistical dependences were significant if their level of significance was *p* < 0.05. The analysis was carried out using Statistica 13.0 PL.

## 3. Results

A comparison was made between the results obtained for the movement task on the horizontal track during exercise from right-to-left (marked in the software as −90), from left-to-right (marked in the software as 90), and on the vertical track, in both the study and control groups for measurements I–III. In the exercise on the horizontal track from right-to-left (Table 2), the differences in the analyzed parameters between the study and control groups were significant across all measurements for COG outside the horizontal track and for COG distance. Statistical relevance was also observed between groups concerning the inside the horizontal track for the first two measurements, and the total time excursion for the first measurement. Statistically important differences between successive measurements were recorded for all parameters analyzed in this exercise.

The COG dwell time inside the horizontal track (t inside) during the left-to-right exercise (90), as well as outside the horizontal track (t outside), the COG distance, and the total time were compared (Table 2). For the first two measurements, the differences between the study and control groups were statistically significant (*p* < 0.001) for COG inside the horizontal track, outside the horizontal track, and total time. Statistically important differences were observed between the analyzed groups across all measurements (I–III) in the case of COG distance. In the case of the remaining analyzed parameters, significantly different results for the compared groups were indicated in the first and second measurements. Considerably relevant differences were noted between successive measurements (I–III) for all analyzed parameters in this exercise.

The average time spent inside the vertical track, outside the vertical track, COG distance, and total COG time in this exercise were subjected to comparative analysis (Table 3). The differences within a group between all measurements were shown to be statistically significant (*p* < 0.001) for all parameters tested. Statistically significant differences were observed between the groups across all measurements of the COG distance in this exercise, and in the last measurement of total time. Statistically relevant differences were observed between consecutive measurements in all the analyzed parameters in this exercise.

**Figure 2:**
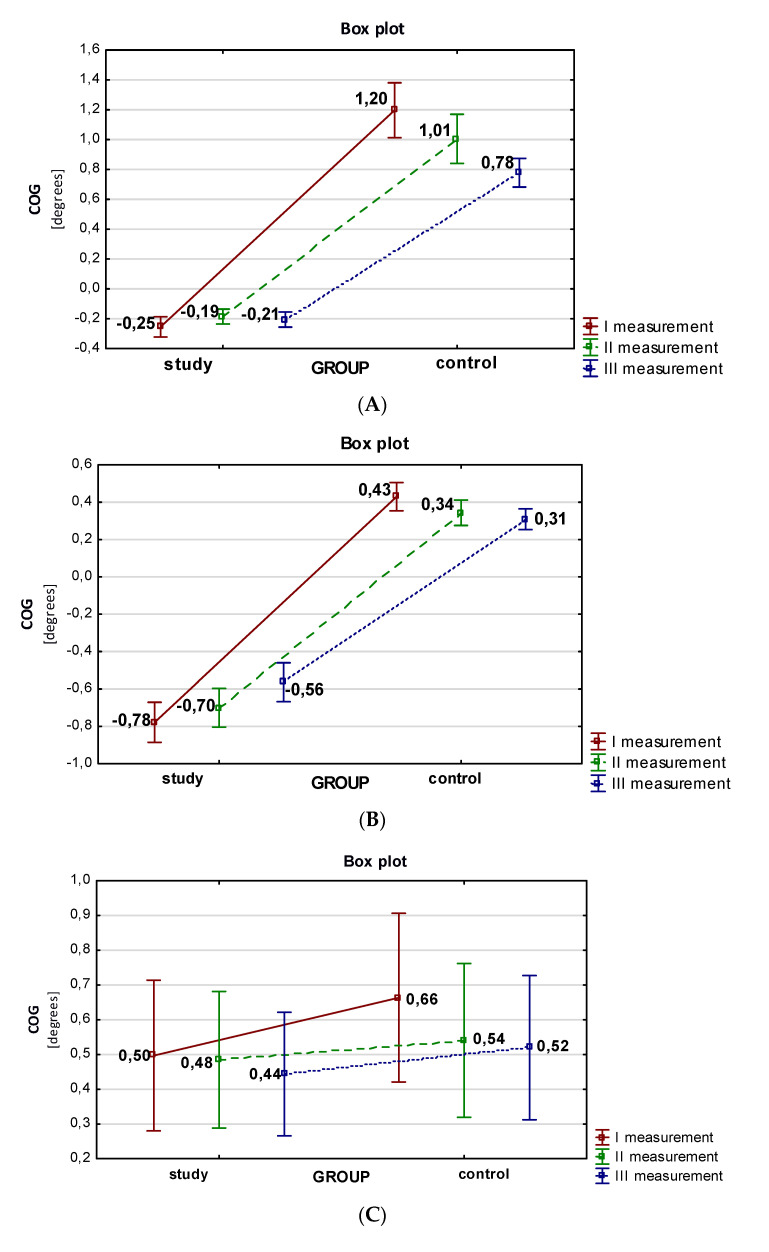

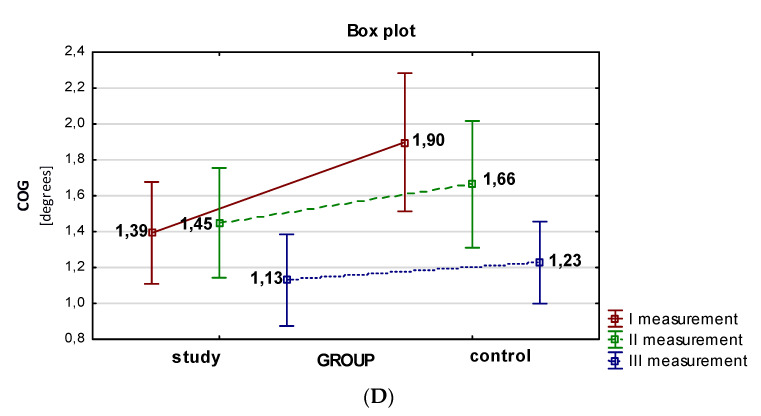
(**A**) Results of COG rightward tilt measurements with eyes closed (right EC test) for the compared groups; (**B**) results of the measurement of COG tilting to the left with eyes closed (left EC test) for the compared groups; (**C**) results of COG tilting measurements (degrees) in the posterior direction with eyes closed (backward EC test) for the compared groups; (**D**) average measurements of COG tilts in the anterior direction with the eyes closed (forward EC test) for the compared groups. COG—center of gravity.

After completing the set of motor exercises, each patient in the study and control groups had their COG excursion registered in four directions (right, left, back, and front) during the tilt test on the Balance Trainer platform. This was performed three times, with the visual control disabled. The average values of the COG tilt to the right with the eyes closed (right EC test) during the balance test indicate that the patients in the study group obtained significantly lower values of the analyzed parameter for each of the measurements, as shown in Figure 2A. In this group, the average value of the analyzed parameter decreased in the second measurement to 0.19, before increasing to −0.21 in the third measurement.

The average measurements of the COG leftward tilt with the eyes closed are shown in Figure 2B. There was a decreasing trend in the results obtained across the successive measurements in both groups. Patients in the control group obtained higher average COG tilts to the left (left EC test) during the balance test for each measurement.

A comparative analysis of the average COG backward excursion (backward EC test) with the eyes closed is shown in Figure 2C. A greater tilting was observed in the control group. In both groups, there was a decrease in backward tilting across the subsequent measurements.

A comparative analysis of the average COG forward tilt (forward EC test) with eyes closed was performed. Patients in the control group achieved greater COG forward tilting with eyes closed (forward EC test) for each measurement (Figure 2D).

To show the functioning of the patients from the study and control groups, the results were analyzed using the functional scales of Brunnström, Ashworth, Rankin, and Barthel. To visualize the level of pain during the four weeks of rehabilitation, the VAS was used. Statistically significant correlations were obtained between groups for all measurements using the Brunnström scale (assessing the functional status of the lower limb) and the Rankin scale (assessing disability level). To diagnose the level of muscle spasticity in post-stroke patients, a three-measurement test was performed using the lower limb Ashworth scale. Statistically significant results between groups were recorded for the third measurement. Statistically relevant results between groups were recorded for the third measurement. To assess the mobility of patients after stroke, the Barthel scale was used. Comparative analysis of the obtained results between groups demonstrated statistically significant differences for the second measurement on this scale; regarding the other two measurements, significant relationships were not found. Considering the results obtained using the VAS, it can be stated that there is a statistically relevant correlation between the groups for the second and third measurements. Statistically significant differences were noted between the consecutive measurements on all analyzed scales (Table 4).

## 4. Discussion

Imbalance occurring in patients after ischemic stroke is a significant therapeutic problem. Modern rehabilitation methods provide a wide variety of improvement programs that significantly enhance brain reorganization processes. One of these methods is biofeedback, used during static balance training, from which stroke patients obtain information regarding the position of the body’s center of gravity (COG) during simple movement tasks, and also learn how to regain balance if they lose it [32,43]. The research of many authors proves considerably greater rehabilitation effects among patients using the biofeedback method [11,13,21,22,23,24,25,26,44]. Treadmill training based on biofeedback offers new possibilities for modern neurorehabilitation, influencing the improvement in gait of stroke patients and triggering defense mechanisms against falls [45,46,47]. An additional 15 min of balance training with visual feedback on the Force platform guarantees greater COG control compared to neurodevelopmental training alone [48]. The literature reports on the use of the Balance Master to assess the functional status of stroke patients using the Barthel index. For example, there was an improvement in functional status in 77.6% of patients receiving feedback training compared to patients receiving conventional therapy alone [49]. At the same time, patients using the MTD Control platform as an additional form of therapy had improved balance and the ability to consciously control lower limb loading [50]. When considering the effectiveness of core exercises for postural control in post-stroke patients, researchers emphasize that conventional training along with additionally guided stimulation of the nervous system achieves the expected results [51]. Significant improvements in post-stroke patients’ body balance have been demonstrated due to the activation of the gluteal muscles and the ability to progressively load the hemiparetic side [52]. Some authors suggest that both rehabilitation procedures contribute to progress in the postural control system of post-stroke patients, and that biofeedback training has additional benefits for balance among patients that used it compared to patients that were rehabilitated without visual biofeedback [13,53,54]. The results presented in [55] show that biofeedback training on a Balance Trainer has a beneficial effect on patients’ balance and posture, although it has not been shown to sufficiently improve functional activities in stroke patients [55]. Other studies also confirm the improvement of static balance and the reduction of spasticity in stroke patients that exercised for four weeks on a Thera-Trainer (a newer version of the Balance Trainer) with the additional application of TENS currents [56]. Ours is the first study using the Balance Trainer device in the proposed movement task scheme. Unlike the aforementioned studies, no physical therapy was used here.

The results of our study indicate a greater improvement in functional status and body balance in patients that exercise with the Balance Trainer device simultaneously with conventional exercises, compared to the control group that exclusively performed conventional exercises. This is reflected in the ability of patients to perform daily activities and improve their independence. We observed changes in the analyzed parameters during the first two weeks of rehabilitation where the study group, in addition to traditional exercises using neurophysiological methods, benefited from exercises on the Balance Trainer platform, while the control group had a set improvement program based only on traditional forms of therapy. Significant improvement was also observed among patients in the control group when additional biofeedback training was introduced. In the literature, there are various reports on the need for a balanced exercise program with visual feedback to be introduced into the rehabilitation of stroke patients [57]. One method is Physiosensing platform exercises, which include exercises to maintain the body’s center of gravity in the sagittal plane and frontal plane at the same time, and dynamic balance exercises that involve moving the COG in multiple directions (movements in a circle, square, and maze paths). There were improvements in dynamic balance, postural control, and symmetry of load distribution in the lower limbs. The improvement in functional status was suggested by an increase of 20 points on the Berg scale, symmetrical load distribution in the frontal and sagittal planes, and a reduction in exercise time from 1.24 min to 0.62 min [57]. Similar results were obtained in our study, where the average total time of the performed exercises decreased when moving the COG on the horizontal track from right-to-left, from left-to-right, and on the vertical track in both groups. In the case of movement on the horizontal track from left-to-right, a slight increase in this parameter was observed for the second measurement in the study group. In the control group, this time decreased during successive recordings of this motor task. The decreasing trend in the total time during the analyzed exercises indicates that the balance of the stroke patients improved. The fastest exercise was the horizontal track movement from right-to-left performed by patients in the control group. In contrast, the greatest difference in the time required to perform a given movement task, compared to the baseline time, was obtained during the vertical track exercises performed by patients in the control group during the last measurement. In this group, the greatest improvement (reduction in time by 58.2 s) in the analyzed parameter was observed, visible after two weeks from the introduction of biofeedback training to the rehabilitation process. Similar results were presented in [58]. After training on the Balance Trainer, the time to perform a movement task was reduced among patients with balance disorders (by 33 s relative to the initial value) and the precision of the patients’ movements improved significantly. The accuracy of the exercises can be observed by analyzing the result of the COG distance, which was shortened by 9.7 cm [58]. In our study, similar to the aforementioned reports, during exercise in the frontal and sagittal plane, the average COG distance decreased compared to the value measured on the day the patients were admitted to the clinic. The shortest COG path (a value 7.99 cm lower than the second measurement) was recorded among patients in the control group during the left-to-right horizontal track exercise in the last measurement. When Balance Trainer exercises were incorporated into the rehabilitation program, patients in this group had the most significant statistical improvement in the symmetric loading of the lower extremities. The control group achieved considerably superior results in terms of the total time required to perform COG exercises on a horizontal and vertical track. A significant improvement in balance, i.e., a reduction in total time during the exercises, was obtained only when training on the Balance Trainer was introduced into the rehabilitation process. Patients in the study group, after four weeks of biofeedback exercise, also showed improvement in lower limb loading, as evidenced by the statistically significant results. However, consideration is due before introducing new exercises after two weeks of performing the same movement tasks. Exercises that are individually adjusted in terms of difficulty for the patients would make them more engaged, performing them faster and more precisely. In this study, patients in the analyzed groups achieved the greatest improvement in lower limb function during two weeks of rehabilitation, which included additional training on the Balance Trainer.

Neurorehabilitation challenges physicians and physiotherapists to assess the clinical condition of patients in a multi-profile and objective manner. Taking these needs into account, several research tools are now being used as measures of functional status. In [32], the effects of treatment of post-stroke patients were assessed using Brunnström and Barthel scales. Patients received the following medical procedures: individual exercises, group exercises, apparatus work, occupational therapy, physical therapy, and psychotherapy. Individual exercises were conducted in the form of passive, assisted, guided, and active exercises, and were based on the latest neurophysiological methods [32]. The study was based on the methodology of research presented in [32]. In our study and control groups, the same set of kinesiotherapeutic exercises were used, i.e., balance exercises, motor coordination, whole-body training (using the PNF and Bobath methods), breathing exercises, and active or passive verticalization, depending on the functional status of patients. The effects of rehabilitation were monitored using the Brunnström and Barthel scales. The Ashworth scale and VAS were also used for a comprehensive assessment. In the case of the study presented in [32], a significant improvement in functional capacity was observed in all patients assessed using the Brunnström (1.14 degree increase) and the Barthel scale (3.25 degree increase). In the present study, similar results were obtained. In both groups, an improvement in the functional status of the patients was observed on the basis of a triple examination. In terms of lower limb function on the Brunnström scale, the study group improved by 0.89 points, and the control group by 1.55 points. This is the difference between the first and third measurements. Patients in the control group started training on the Balance Trainer after two weeks of conventional rehabilitation. Therefore, additional biofeedback exercises were only conducted for 10 days. Thus, the difference between the second and third measurements obtained by patients in the control group is 1.28 degrees, indicating a more distinct improvement in the functional status of patients within this group. In the study group, after two weeks of rehabilitation, an improvement in the function of the paresis of the lower limb evaluated according to the Brunnström scale was found to be 0.44 degrees. However, in the control group, the recorded improvement was 0.27 degrees. Therefore, the effects of rehabilitation were similar in both study groups; however, the group with Balance Trainer exercises showed greater improvement.

Similar results were obtained in the study presented in [33]. The group of post-stroke patients using biofeedback training improved by 0.54 degrees, while the control group improved by 0.48 degrees. A factor that may influence the results is the patients’ initial functional status. Faster improvement was obtained among patients that had very severe impairments in the symmetrical loading of the lower limbs [33]. The average Barthel scale scores in our experiment suggest a significant improvement in activities of daily living. The differences between the first and second measurements in the study and control groups were 10.96 points and 6.99 points. The values of the average results on this scale were higher in the case of the study group, which may be caused by the introduction of the biological feedback exercises into the rehabilitation program. It has been reported that training on the Tecnobody Stability Easy platform can be used as an additional form of therapy that, when combined with comprehensive rehabilitation, increases balance control in post-stroke patients, which influences the final results of rehabilitation as expressed by Barthel [59].

In the study of [60], it was indicated that rehabilitation involving biofeedback devices, with the use of motion sensors and a platform with barometric pressure sensors, definitely improves motor skills and balance by motivating patients to exercise.

In the present study, the tilt test was performed three times on the patients. Before proceeding to the medical experiment, we asked ourselves what role the tilt test was intended to fulfil in the light of this study, diagnostic or therapeutic? In the case of free- standing with both feet and the arms extended in front of the patient with the eyes open, to obtain the best possible result by maintaining the most upright position, the patient performed a large compensation with the trunk towards the paresis lower limb. The posture was unstable, and the patients were unable to maintain a standing position for 10 s with their eyes open. When visual control was excluded, the balance system lost the additional signal that informs on body position, working under greater stress and more efficiently. Although the test performed by patients with eyes closed was more difficult to perform due to hemiparesis, patients were able to demonstrate a greater focus, and this test provided valuable and objective information on the position of the COG during independent standing with both feet among post-stroke patients. The literature reports that the amplitude of horizontal body tilt is a reliable measure of balance. It indicates that the greater the tilt, the worse the balance [61]. When measuring the effect of exercise using biological feedback on the dynamic balance of stroke patients, a highly statistically significant difference (*p* < 0.001) was found in the symmetry index values after three weeks of rehabilitation between patients exercising and not exercising on a balance platform [62]. Our study confirms the above medical reports. The improvement in the symmetry of the loading of the right and left limb should be attributed to the results of the lateral tilting of the COG. In the present experiment, significantly higher values of the lateral tilting of the COG in all directions during the analyzed test in measurements I–III were recorded in the control group. The patients in this group exercised on the Balance Trainer in addition to the conventional rehabilitation for two weeks, from Monday to Friday, i.e., 10 times. The study group, on the other hand, conducted additional training for four weeks (20 exercises). Complementing the basic rehabilitation with training on a balance platform using biological feedback affects the reduction of COG in all directions, which is reflected in the diagnostic test performed. In our study, both in the study group and control group, the highest value was recorded for forward tilting; alternatively, the lowest value was for right tilting in the study group, and left tilting in the control group. Considerably different values were obtained in the study of [33], using the Balance Trainer device among post-stroke patients. The highest COG excursions in both groups were recorded towards the back. On the other hand, the smallest was in the forward direction. Moreover, both groups presented an improvement in the static balance of the body, and in the group in which Balance Trainer platform exercises were conducted, significantly lower values of tilting were obtained [33]. In our study, a rehabilitation program in which additional exercises on the Balance Trainer device were used, brought a greater therapeutic effect in the form of improvement of static body balance. Despite the decrease in the value of tilting in all directions, there was still a predominance of forward tilting in both groups. Similar results were obtained by [63]. The symmetry of loading of the lower limbs was improved. The speed and amplitude of body tilting of patients with lateral and anterior–posterior hemiparesis did not significantly decrease until four weeks after the application of biological feedback. Improvements began to occur two weeks after admission. Fewer COG excursions were obtained in the lateral directions (right, left) than in the anteroposterior direction [63]. The literature data highlight that load asymmetry decreased during the first four weeks, with no further improvement thereafter [63,64].

Our research indicates that the patients’ balance is improved by the average value of the distance covered by the COG during the exercises on the horizontal track (from right-to-left and vice versa) and on the vertical track (from back-to-front). The values in the analyzed groups decreased over the three measurements, so that the patients performed the exercise more precisely, putting more strain on the side with paresis.

To illustrate the level of pain intensity of patients in the study and control groups, the visual analogue pain scale (VAS) was used in our study. After two weeks of rehabilitation, the patients in the study group reported slightly less pain (1.17 points less than at the beginning of therapy). When presenting the differences in further measurements, a significant decrease in pain intensity was obtained in the control group. After two weeks of introducing exercises on the Balance Trainer device, pain decreased among patients by 2.9 points in this group. In comparison, the study group achieved a 1.25 point reduction in pain at the final examination. A significant improvement in this regard was therefore obtained in the control group, after two weeks of exercise using biological feedback. Slightly different results were presented in a previous study [63]. 

Our research confirms medical reports that training with the use of biological feedback influences the improvement of body balance, lower limb function, and control in loading of the paresis side. The progress of rehabilitation in this study was measured using Brunnström, Barthel, Rankin, and Ashworth scales, which provided information on the functional status of post-stroke patients. 

## 5. Conclusions

The analysis shows that supplementing the basic rehabilitation program with training on the Balance Trainer reduces COG tilts in all directions during relaxed standing with the visual control excluded. It should be added that the training with the use of biological feedback, applied to patients after stroke, reduces the degree of spasticity, improves the function of the lower limb, and reduces the degree of disability and pain. What seems to be crucial is that the training with the use of biological feedback also conditions an increase in the independence among these individuals. After the application of the mentioned balance training, the precision of the exercises improved (reduction in the time spent out of the area, reduction in the total time of the exercises, reduction in the COG distance). 

## Figures and Tables

**Table 1:** Characteristics for the study and control groups.

Variable	Study Group (*n* = 92)	Control Group (*n* = 92)	Study + Control Group (*n* = 184)
Sex (male/female)	54/38	51/41	105/79
* Age (in years)	62.00 (7.23)	63.14 (6.51)	62.57 (6.88)
*$\;\mathrm{BMI}\;(\frac{\mathrm{kg}}{m^{2}}$)	27.22 (2.23)	26.20 (1.81)	26.71 (2.09)
Paretic side (left/right)	36/56	71/21	107/77

* presented as mean (X) and standard deviation (SD) of the parameters.

**Table 2:** Summary of results for the horizontal track right-to-left exercise (−90) and summary of results for the left-to-right horizontal track movement task (90).

Exercise on a Horizontal Track from Right-to-Left							
Group	I		II		III		*p* *
	X	SD	X	SD	X	SD	
Time (t inside) of COG inside the horizontal track (s)							
Study group	36	23.4	31.2	20.4	28.2	20.4	<0.001
Control group	48	27.6	36	24	24.6	14.4	<0.001
*p* **	<0.001		0.019		0.788		
Time (t outside) of the COG outside the horizontal track (s)							
Study group	7.2	9.6	6.6	10.2	3.6	6	<0.001
Control group	9	9.6	6	4.8	3	3	<0.001
*p* **	<0.001		0.010		0.050		
COG distance (cm)							
Study group	28.96	10.08	27.84	10.55	22.85	6.98	<0.001
Control group	35.62	14.18	35.03	13.11	27.52	8.80	<0.001
*p* **	<0.001		<0.001		<0.001		
Total time (s)							
Study group	43.2	25.2	37.2	23.4	31.8	21.6	<0.001
Control group	57	31.2	42	25.2	27.6	15	<0.001
*p* **	<0.001		0.119		0.936		
Exercise on a horizontal track from left-to-right							
Time (t inside) of COG inside the horizontal track (s)							
Study group	37.2	24.6	49.8	172.8	28.8	21.6	<0.001
Control group	70.8	225	38.4	22.2	25.8	17.4	<0.001
*p* **	<0.001		0.001		0.536		
Time (t outside) of the COG outside the horizontal track (s)							
Study group	16.8	81	13.8	81.6	12.6	69.6	<0.001
Control group	12	11.4	7.2	6.6	4.2	4.2	<0.001
*p* **	0.002		0.001		0.108		
COG distance (cm)							
Study group	29.98	9.552	26.15	6.872	24.66	6.608	<0.001
Control group	38.16	13.72	37.36	13.08	29.37	12.17	<0.001
*p* **	<0.001		<0.001		0.001		
Total time (s)							
Study group	54.0	83.4	63.6	190.2	41.4	70.2	<0.001
Control group	82.8	224.4	45.6	23.4	30.6	17.4	<0.001
*p* **	<0.001		0.002		0.499		

I—Measurement I; II—Measurement II; III—Measurement III; X—mean (min); SD—standard deviation; *p* *—level of significance between measurements (Friedmann’s ANOVA); *p* **—level of significance between groups (Mann–Whitney U test).

**Table 3:** Summary of results for the vertical track movement task.

Exercise on a Vertical Track							
Group	I		II		III		*p* *
	X	SD	X	SD	X	SD	
Time (t inside) of COG inside the horizontal track (s)							
Study group	48	36	37.2	25.8	32.4	21.6	<0.001
Control group	80.4	337.8	36.6	30.6	27	24	<0.001
*p* **	0.921		0.894		0.097		
Time (t outside) of the COG outside the horizontal track (s)							
Study group	7.8	13.8	16.8	112.8	13.2	88.2	<0.001
Control group	6.6	9	4.2	6	2.4	3.6	<0.001
*p* **	0.053		0.055		0.491		
COG distance (cm)							
Study group	28.0	9.54	26.1	8.75	22.5	7.36	<0.001
Control group	30.94	11.90	29.69	11.01	25.06	9.57	<0.001
*p* **	0.033		0.013		0.035		
Total time (s)							
Study group	55.8	39	54	114.6	45.6	89.4	<0.001
Control group	87	337.8	40.8	31.8	28.8	24	<0.001
*p* **	0.611		0.433		0.013		

I—Measurement I; II—Measurement II; III—Measurement III; X—mean (min); SD—standard deviation; *p* *—level of significance between measurements (Friedmann’s ANOVA); *p* **—level of significance between groups (Mann–Whitney U test).

**Table 4:** Summary of patients’ functional status scores.

Brunnström scale, lower limb							
Group	I		II		III		*p* *
	X	SD	X	SD	X	SD	
Study group	3.48	1.02	3.92	0.85	4.37	0.75	<0.001
Control group	3.12	1.00	3.39	0.95	4.67	0.71	<0.001
*p* **	0.037		0.001		0.009		
Ashworth scale, lower limb							
Study group	1.76	0.91	1.50	0.65	1.26	0.57	<0.001
Control group	1.59	0.73	1.47	0.70	0.64	0.64	<0.001
*p* **	0.274		0.689		<0.001		
Rankin scale							
Study group	2.30	1.09	1.99	0.87	1.72	1.36	<0.001
Control group	2.86	0.83	2.62	0.77	1.99	0.72	<0.001
*p* **	0.001		< 0.001		0.003		
Barthel scale							
Study group	50.90	17.40	61.86	15.97	71.42	14.99	<0.001
Control group	46.79	12.61	53.78	12.59	67.57	10.77	<0.001
*p* **	0.491		0.002		0.050		
VAS							
Study group	7.27	1.53	6.10	1.10	4.85	0.97	<0.001
Control group	7.62	1.12	6.52	1.11	3.62	1.19	<0.001
*p* **	0.244		0.013		<0.001		

I—Measurement I; II—Measurement II; III—Measurement III; X—mean (min); SD—standard deviation; *p* *—level of significance between measurements (Friedmann’s ANOVA); *p* **—level of significance between groups (Mann–Whitney U test).

## Data Availability

Data available on request.

## References

[1] Johnson C.O., Nguyen M., Roth G.A., Nichols E., Alam T., Abate D., Abd-Allah F., Abdelalim A., Abraha H.N., Abu-Rmeileh N. (2019). Global, regional, and national burden of stroke, 1990–2016: A systematic analysis for the Global Burden of Disease Study 2016. Lancet Neurol..

[2] Abubakar I.I., Tillmann T., Banerjee A. (2015). Global, regional, and national age-sex specific all-cause and cause-specific mortality for 240 causes of death, 1990–2013: A systematic analysis for the Global Burden of Disease Study 2013. Lancet.

[3] Mathers C.D., Loncar D. (2006). Projections of global mortality and burden of disease from 2002 to 2030. PLoS Med..

[4] De Nunzio A.M., Zucchella C., Spicciato F., Tortola P., Vecchione C., Pierelli F., Bartolo M. (2014). Biofeedback rehabilitation of posture and weight-bearing distribution in stroke: A center of foot pressure analysis. Funct. Neurol..

[5] Donkor E.S. (2018). Stroke in the 21(st) Century: A Snapshot of the Burden, Epidemiology, and Quality of Life. Stroke Res..

[6] Hugues A., Di Marco J., Ribault S., Ardaillon H., Janiaud P., Xue Y., Zhu J., Pires J., Khademi H., Rubio L. (2019). Limited evidence of physical therapy on balance after stroke: A systematic review and meta-analysis. PLoS ONE.

[7] Tyson S.F., Hanley M., Chillala J., Selley A., Tallis R.C. (2006). Balance disability after stroke. Phys. Ther..

[8] Bowman T., Gervasoni E., Arienti C., Lazzarini S.G., Negrini S., Crea S., Cattaneo D., Carrozza M.C. (2021). Wearable Devices for Biofeedback Rehabilitation: A Systematic Review and Meta-Analysis to Design Application Rules and Estimate the Effectiveness on Balance and Gait Outcomes in Neurological Diseases. Sensors.

[9] Hasegawa N., Asaka T. (2021). Motor learning on postural control using auditory biofeedback training. Impact.

[10] Zhang X., Yue Z., Wang J. (2017). Robotics in Lower-Limb Rehabilitation after Stroke. Behav. Neurol..

[11] Tiseo C., Lim Z.Y., Shee C.Y., Ang W.T. (2014). Mobile Robotic Assistive Balance Trainer—An intelligent compliant and adaptive robotic balance assistant for daily living. Proceedings of the 2014 36th Annual International Conference of the IEEE Engineering in Medicine and Biology Society.

[12] Burgos P.I., Lara O., Lavado A., Rojas-Sepúlveda I., Delgado C., Bravo E., Kamisato C., Torres J., Castañeda V., Cerda M. (2020). Exergames and Telerehabilitation on Smartphones to Improve Balance in Stroke Patients. Brain Sci..

[13] Tieri G., Morone G., Paolucci S., Iosa M. (2018). Virtual reality in cognitive and motor rehabilitation: Facts, fiction and fallacies. Expert Rev. Med. Devices.

[14] Ghanbari Ghoshchi S., De Angelis S., Morone G., Panigazzi M., Persechino B., Tramontano M., Capodaglio E., Zoccolotti P., Paolucci S., Iosa M. (2020). Return to Work and Quality of Life after Stroke in Italy: A Study on the Efficacy of Technologically Assisted Neurorehabilitation. Int. J. Environ. Res. Public Health.

[15] Morone G., Paolucci S., Cherubini A., De Angelis D., Venturiero V., Coiro P., Iosa M. (2017). Robot-assisted gait training for stroke patients: Current state of the art and perspectives of robotics. Neuropsychiatr. Dis. Treat..

[16] Lledó L.D., Díez J.A., Bertomeu-Motos A., Ezquerro S., Badesa F.J., Sabater-Navarro J.M., García-Aracil N. (2016). A Comparative Analysis of 2D and 3D Tasks for Virtual Reality Therapies Based on Robotic-Assisted Neurorehabilitation for Post-stroke Patients. Front. Aging Neurosci..

[17] Slater M., Sanchez-Vives M.V. (2016). Enhancing our lives with immersive virtual reality. Front. Robot. AI.

[18] Giggins O.M., Persson U.M., Caulfield B. (2013). Biofeedback in rehabilitation. J. Neuro. Eng. Rehabil..

[19] Pollock A., Farmer S.E., Brady M.C., Langhorne P., Mead G.E., Mehrholz J., Van Wijck F. (2014). Interventions for improving upper limb function after stroke. Cochrane Database Syst. Rev..

[20] Zakharov A.V., Bulanov V.A., Khivintseva E.V., Kolsanov A.V., Bushkova Y.V., Ivanova G.E. (2020). Stroke Affected Lower Limbs Rehabilitation Combining Virtual Reality With Tactile Feedback. Front. Robot. AI.

[21] Jeon H.J., Hwang B.Y. (2018). Effect of bilateral lower limb strengthening exercise on balance and walking in hemiparetic patients after stroke: A randomized controlled trial. J. Phys. Ther. Sci..

[22] Jung K.S., Cho H.Y., In T.S. (2016). Trunk exercises performed on an unstable surface improve trunk muscle activation, postural control, and gait speed in patients with stroke. J. Phys. Ther. Sci..

[23] Jung K., Kim Y., Chung Y., Hwang S. (2014). Weight-shift training improves trunk control, proprioception, and balance in patients with chronic hemiparetic stroke. Tohoku J. Exp. Med..

[24] Park Y.K., Kim J.H. (2017). Effects of kinetic chain exercise using EMG-biofeedback on balance and lower extremity muscle activation in stroke patients. J. Phys. Ther. Sci..

[25] Tamburella F., Moreno J.C., Valenzuela D.S.H., Pisotta I., Iosa M., Cincotti F., Mattia D., Pons J.L., Molinari M. (2019). Influences of the biofeedback content on robotic post-stroke gait rehabilitation: Electromyographic vs joint torque biofeedback. J. Neuroeng. Rehabil..

[26] Cho J.E., Yoo J.S., Kim K.E., Cho S.T., Jang W.S., Cho K.H., Lee W.H. (2018). Systematic review of appropriate robotic intervention for gait function in subacute stroke patients. Biomed Res. Int..

[27] Leightley D., Hoon M.Y., Coulson J., Piasecki M., Cameron J., Barnouin Y., Tobias J., McPhee J.S. (2017). Postural stability during standing balance and sit-to-stand in master athlete runners compared with nonathletic old and young adults. J. Aging Phys. Act..

[28] Winiarska A., Ziółkowska A., Świtaj K., Wojtczak P. (2017). Balance of individuals at different age involved in physical activity—review of publications. J. Educ. Health Sport.

[29] Fung J. (2017). Gait and balance training using virtual reality is more effective for improving gait and balance ability after stroke than conventional training without virtual reality. J. Physiother..

[30] Krekora K., Czernicki J. (2005). Biofeedback in rehabilitation of stroke patients. Med. Rehabil..

[31] Huang H., Wolf S.L., He L. (2006). Recent developments in biofeedback for neuromotor rehabilitation. J. Neuroeng. Rehabil..

[32] Przysada G., Guzik A., Wolan-Nieroda A., Walicka-Cupryś K., Drużbicki M. (2015). Chosen assessment methods of physiotherapy effects in patients after cerebral stroke treated at a rehabilitation ward. Med. Rev..

[33] Gałęcki S., Walasik M., Rokicki R., Sikorska K., Dudkiewicz Z. (2013). Effectiveness of rehabilitation and exercises on static-dynamic parapodium with biofeedback in relation to body balance in patients after ischaemic stroke. Kwart. Ortop..

[34] Jung K.W., Yang D.H., Myung S.J., Ramachandran V.S. (2012). Biofeedback therapy. The Encyclopedia of Human Behawior.

[35] Glick R.M., Greco C.M. (2010). Biofeedback and primary care. Prim. Care.

[36] Genthe K., Schenck C., Eicholtz S., Zajac-Cox L., Wolf S., Kesar T.M. (2018). Effects of real-time gait biofeedback on paretic propulsion and gait biomechanics in individuals post-stroke. Top. Stroke Rehabil..

[37] Kiper P., Agostini M., Luque-Moreno C., Tonin P., Turolla A. (2014). Reinforced feedback in virtual environment for rehabilitation of upper extremity dysfunction after stroke: Preliminary data from a randomized controlled trial. Biomed Res. Int..

[38] Kiper P., Baba A., Agostini M., Turolla A., Kiper A., Nowobilski R., Opara J., Szczudlik A. (2017). Motor learning and brain plasticity after stroke. State of the art. Rehabil. W Prakt..

[39] Kawato M. (1999). Internal models for motor control and trajectory planning. Curr. Opin. Neurobiol..

[40] Hung J.W., Yu M.Y., Chang K.-C., Lee H.-C., Hsieh Y.-W., Chen P.-C. (2016). Feasibility of Using Tetrax Biofeedback Video Games for Balance Training in Patients with Chronic Hemiplegic Stroke. PM&R.

[41] Brennan L., Zubiete E.D., Caulfield B. (2020). Feedback Design in Targeted Exercise Digital Biofeedback Systems for Home Rehabilitation: A Scoping Review. Sensors.

[42] Reinkensmeyer D., Burdet E., Casadio M., Krakauer J., Kwakkel G., Lang C., Swinnen S., Ward N., Schweighofer N. (2016). Computational neurorehabilitation: Modeling plasticity andlearning to predict recovery. J. Neuroeng. Rehabil..

[43] Bulat T., Hart-Hughes S., Ahmed S., Quigley P., Palacios P., Werner D.C., Foulis P. (2007). Effect of a group-based exercise program on balance in elderly. Clin. Interv. Aging.

[44] Maciaszek J. (2018). Effects of posturographic platform biofeedback training on the static and dynamic balance of older stroke patients. J. Stroke Cerebrovasc. Dis..

[45] Begg R. (2018). Can Real-time Biofeedback of Foot Clearance Data be used to Assist with Gait Rehabilitation following Stroke? NHMRC. Impact.

[46] Nagano H., Said C.M., James L., Begg R.K. (2020). Feasibility of Using Foot–Ground Clearance Biofeedback Training in Treadmill Walking for Post-Stroke Gait Rehabilitation. Brain Sci..

[47] Skvortsov D.V., Kaurkin S.N., Ivanova G.E. (2021). A Study of Biofeedback Gait Training in Cerebral Stroke Patients in the Early Recovery Phase with Stance Phase as Target Parameter. Sensors.

[48] Yavuzer G., Eser F., Karakus D., Karaoglan B., Stam H.J. (2006). The effects of balance training on gait late after stroke: A randomized controlled trial. Clin. Rehabil..

[49] Srivastava A., Taly A.B., Gupta A., Kumar S., Murali T. (2009). Post-stroke balance training: Role of force platform with visual feedback technique. J. Neurol. Sci..

[50] Kołcz-Trzęsicka A., Żurowska A., Bidzińska G., Piesiewicz-Białas K., Kobylańska M., Paprocka-Borowicz M. (2017). Use of biofeedback in rehabilitation process of patients after stroke. Med. Sport/Polish J. Sports Med..

[51] Cabrera-Martos I., Ortiz-Rubio A., Torres-Sánchez I., López-López L., Jarrar M., Valenza M.C. (2020). The effectiveness of core exercising for postural control in patients with stroke: A systematic review and meta-analysis. PM&R.

[52] Pak N.W., Lee J.H. (2020). Effects of visual feedback training and visual targets on muscle activation, balancing, and walking ability in adults after hemiplegic stroke: A preliminary, randomized, controlled study. Int. J. Rehabil. Res..

[53] Ghomashchi H. (2016). Investigating the effects of visual biofeedback therapy on recovery of postural balance in stroke patients using a complexity measure. Top. Stroke Rehabil..

[54] Koroleva E.S., Kazakov S.D., Tolmachev I.V., Loonen A.J.M., Ivanova S.A., Alifirova V.M. (2021). Clinical Evaluation of Different Treatment Strategies for Motor Recovery in Poststroke Rehabilitation during the First 90 Days. J. Clin. Med..

[55] Ordahan B., Karahan A.Y., Basaran A., Turkoglu G., Kucuksarac S., Cubukcu M., Tekin L., Polat A.D., Kuran B. (2015). Impact of exercises administered to stroke patients with balance trainer on rehabilitation results: A randomized controlled study. Hippokratia.

[56] Yanga Y., Leeb J., Choic W., Joob Y., Lee S. (2020). Balance trainer training with transcutaneous electrical nerve stimulation improves spasticity and balance in persons with chronic stroke. Phys. Ther. Rehabil. Sci..

[57] Ventura A., Mendes J., Caldeira R., Pinto S., Pereira Â.M. Benefit of balance retraining after stroke using a force platform biofeedback—Case Study. Proceedings of the 2nd International Congress of CiiEM: Translational Research and Innovation in Human and Health Science.

[58] Bałdyga E., Białkowska J. The use of biofeedback in patients with neurological deficits. Proceedings of the Materiały zjazdowe, Conference V Olsztyński Dzień Fizjoterapii.

[59] Jankowska A., Klimkiewicz P., Krukowska S., Woldańska-Okońska M. (2021). Assessment of the Impact of Training on the Stabilometric Platform Using the Biofeedback Method on Improving Balance and Functional Efficiency of Patients After a Stroke. Acta Balneol..

[60] Lupo A., Cinnera A.M., Pucello A., Iosa M., Coiro P., Personeni S., Gimigliano F., Iolascon G., Paolucci S., Morone G. (2018). Effects on balance skills and patient compliance of biofeedback training with inertial measurement units and exergaming in subacute stroke: A pilot randomized controlled trial. Funct. Neurol..

[61] Kuczyński M., Podbielska M.L., Bieć D., Paluszak A.K.K., Kręcisz K. (2012). The basics of postural control assessment: What, how and why do we need to measure. Acta Biooptica Inf. Med..

[62] Bugajski M., Czernicki J. (2013). Evaluation effectiveness of exercises on the balance platform for using biofeedback to walking function in patients after stroke. Medical. Reviev..

[63] de Haart M., Geurts A.C., Huidekoper S.C., Fasotti L., van Limbeek J. (2004). Recovery of standing balance in postacute stroke patients: A rehabilitation cohort study. Arch. Phys. Med. Rehabil..

[64] Yanohara R., Teranishi T., Tomita Y., Tanino G., Ueno Y., Sonoda S. (2014). Recovery process of standing postural control in hemiplegia after stroke. J. Phys. Ther. Sci..

